# Anti-tumor and antioxidant activity of kaempferol-3-O-alpha-L-rhamnoside (Afzelin) isolated from *Pithecellobium dulce* leaves

**DOI:** 10.1186/s12906-022-03633-x

**Published:** 2022-06-22

**Authors:** Masuma Akter, Mst. Shahnaj Parvin, Md. Mahadi Hasan, Md. Aziz Abdur Rahman, Md. Ekramul Islam

**Affiliations:** Department of Pharmacy, Faculty of Science, University of Rajshahi, Rajshahi, 6205 Bangladesh

**Keywords:** Antioxidant, Anti-tumor, EAF, NMR analysis, AAPH, EAC cell

## Abstract

**Background:**

*Pithecellobium dulce* (Roxb.), an evergreen medium-sized, spiny tree which have vast nutritional values and widely used in ayurvedic medicines and home remedies. The plant has also been a rich source of biologically active compounds. The present study was designed to isolate pure compound from ethyl acetate fraction of methanol extract of leaves and to know the efficacy as antioxidant as well as its anti-tumor activity on Ehrlich ascites carcinoma cell (EAC).

**Methods:**

The leaves were extracted with methanol and fractionated with different solvents. The isolation of the compound was carried out by column chromatography from ethyl acetate fraction (EAF) and structure was revealed by ^1^H-NMR and ^13^C NMR. The antioxidant activity was investigated by the scavenging of 2,2-diphenyl-1-picrylhydrazyl (DPPH) free radicals as well as the inhibition of oxidative damage of pUC19 plasmid DNA, hemolysis and lipid peroxidation induced by a water-soluble free radical initiator 2,2’-azo (2-asmidinopropane) dihydrochloride (AAPH) in human erythrocytes. In vivo anti-tumor activity of the compound was also evaluated by determining the viable tumor cell count, hematological profiles of experimental mice along with observing morphological changes of EAC cells by fluorescence microscope.

**Results:**

The isolated compound kaempferol-3-O-alpha-L-rhamnoside effectively inhibited AAPH induced oxidation in DNA and human erythrocyte model and lipid per oxidation as well as a stronger DPPH radical scavenging activity. In anti-tumor assay, at a dose of 50 mg/kg body weight exhibit about 70.89 ± 6.62% EAC cell growth inhibition, whereas standard anticancer drug vincristine showed 77.84 ± 6.69% growth inhibition.

**Conclusion:**

The compound may have a great importance as a therapeutic agent in preventing oxidative damage of biomolecules and therapeutic use in chemotherapy.

**Supplementary Information:**

The online version contains supplementary material available at 10.1186/s12906-022-03633-x.

## Background

Wide variety of chemical compounds synthesized by plants may have important biological functions with defend against attack from predators such as insects, fungi and herbivorous mammals. Many of these phytochemicals have beneficial effects on long-term health when consumed by humans and can be used to effectively treat human diseases. It is well known that the chemical compounds of plants affect the human body by the same process as the chemical compounds of conventional medicine thus herbal medicines do not differ greatly from conventional drugs in terms of how they work. This enables herbal medicines as effective as conventional medicines, but they are less likely to cause harmful side effects [1]. Various studies have reported that medicinal plants are the sources of many nutrient and non-nutrient molecules that could have antioxidant, anti-inflammatory, and antimicrobial activities [2].

Oxidative stress caused by free radical and reactive oxygen species is associated with various diseases. A number of studies have been conducted worldwide to find natural antioxidants in plant sources. Plants containing phenolic and flavonoid compounds have been shown to have strong antioxidant activity. Natural plant-derived products such as flavonoids, terpenes, and alkaloids have received considerable attention in recent years due to their various pharmacological properties, including cytotoxic and cancer chemo protective effects [3].

About 50% of the drugs used for clinical trials were isolated from natural sources. DNA is present in every cells of body and human DNA remains continuously in exposure to free radicals attack that causes damage to DNA. Researchers all over the world are working on the aspect of relating the changes in DNA with evolutionary development [4–6]. Currently, there has been a growing interest in identifying free radical scavengers or antioxidants that prevent DNA from oxidative damage [7]. Another molecule, erythrocytes are considered the main target of free radical attack due to the high membrane concentration of polyunsaturated fatty acids [8]. Therefore, due to their sensitivity to oxidation, erythrocytes have been used as a cellular model to investigate oxidative damage in bio membranes.

The plant *Pithecellobium dulce* belongs to the Fabaceae family and widely distributed in India, Huawei, and tropical Africa and especially along the coast. It has a long history of use in traditional system of medicine. The compound that was isolated from the leaves is a flavonoid glycosides (Kaempferol-3-O-alpha-L-rhamnoside) inhibits lipid peroxidation and cyclooxygenase (COX)-1 and COX-2. Several recent studies have indicated that the compound Kaempferol-3-O-alpha-L-rhamnoside inhibits the growth of breast cancer cells by stimulating apoptosis and that it is relatively non-toxic to normal cells [9]. Ehrlich ascites carcinoma (EAC) is a rapidly growing experimental tumor with very aggressive behavior and resembles human tumors [10]. The potent inhibitory effects of many isolated compounds and crude etracts of different medicinal plants against EAC have also been reported in literature [11]. However, the effects of this compound on the AAPH induced oxidative damage of erythrocytes and pUC19 DNA and also anti-tumor activity against EAC cell has not been investigated.

In this study, we aimed to investigate the effect of kaempferol-3-O-alpha-L-rhamnoside on antioxidant by employing various test models like DPPH scavenging, DNA and erythrocytes damage protection and to check the anti-tumor activity of this compound against EAC cell.

## Methods

### Collection of plant materials

The plant material was collected by following the ethics standard for research activity on plants established at Department of Pharmacy, University of Rajshahi, Bangladesh. Fresh samples of *P. dulce* leaves were collected from the relevant area of Kumarkhali, Kushtia in the months of November-2017 with permission of the owner of the plants and were authenticated by a taxonomist, Prof. AHM Mahbubur Rahman, Dept. of Botany, University of Rajshahi, Bangladesh. A voucher specimen with accession no. 48085 was preserved in National herbarium Dhaka, Bangladesh.

### Ethics of experimentation

The protocol for using human blood cells as well as mice for anti-tumor study was approved by the Institutional Animal, Medical Ethics, Bio-Safety and Bio-Security Committee (IAMEBBC) for Experimentations on Animal, Human, Microbes, and Living Natural Sources at University of Rajshahi (Approval memo no-82/320/IAMEBBC/IBSc, 20 August, 2018). All procedures of this study adhere to the ARRIVE Guidelines for reporting animal research.

### Chemicals and reagents

2,2′-diphenyl-1-picrylhydrazyl (DPPH^•^), 2,2’- Azobis (2- amidinopropane) dihydrochloride (AAPH), catechin, 4,6-diamidino-2-phenylindole (DAPI) (Sigma-Aldrich), Dimethyl sulfoxide; DMSO (E-Merck, Germany)**,** Trypan blue (Sigma, India), Phosphate buffer saline (PBS), pUC19 DNA (Thermo scientific) and Ethidium bromide (Etbr) (Sigma, India).

### Extraction and fractionation

The collected healthy leaves were washed with tap water and dried at room temperature for about 15 days and pulverized by mechanical grinder into coarse powder. Since leaves are rich in chlorophyll so before final extraction about 1.5 kg of leaves powder was first extracted with n-hexane and was discarded since the extract contain chlorophyll. The residue after n-hexane extraction soaked in 3.5 L of methanol and kept it for 12 days with occasional shaking and stirring. The whole mixture was then filtered through cotton and then through Whatmann No.1 filters paper and was concentrated with a rotary evaporator under reduced pressure at 45 °C to collect the crude methanolic extract (CME) and fractionated towards polarity using the solvent n-hexane, chloroform and ethyl acetate. Here, again the n-hexane was used to completely remove the chlorophylls, low polar compounds and fatty materials and was discarded. Other fractions were collected separately and concentrated by rotary evaporation under vacuum.

### Isolation and characterization of the compound

**Fig. 1:**
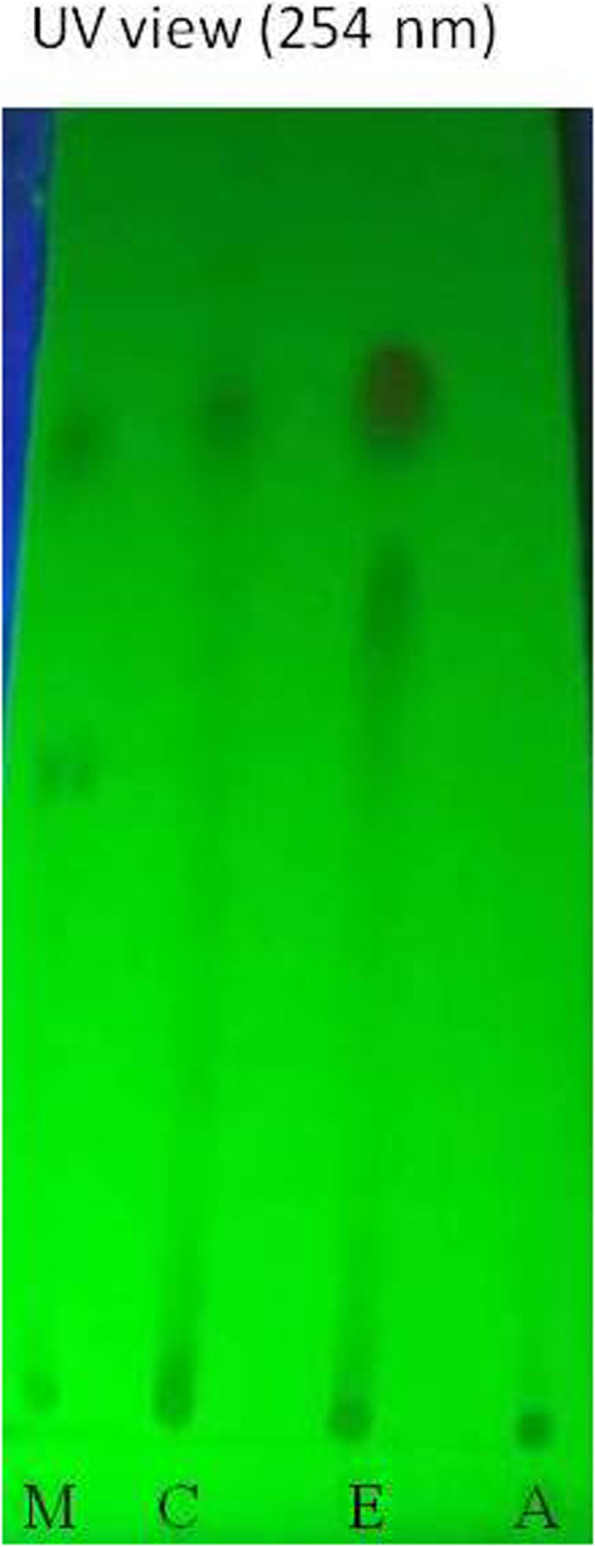
TLC profile at UV 254 nm of methanolic extract and its fraction (CHF, EAF and AQF) of leaf of *P. dulce*. M-Crude methanol extract; C-Chloforform fraction; E-Ethylacetate fraction; A-Aqueous fraction

In vitro biological assay, EAF had potent activity than other fraction and TLC assay also showed some distinct and prominent spot (Fig. 1). Thus, later purification and isolation of active constituents were focused on EAF. This fraction was subjected a silica gel 60 column chromatography using n-hexane: ethyl acetate: methanol (1:2:1) and then ethyl acetate:n-hexane (95:5) as the eluent. Fractions 152 to170 was combined due to similar spot on TLC plate. Then fraction 152 to170 was further purified by preparative thin layer chromatography (PTLC) to isolate the target compound (1.2 g, Rf = 0.65). The compound was dissolved either in methanol and ethyl acetate depending on its solubility for analysis. The structure of the isolated compound was elucidated from the data obtained from ^1^H- and ^13^C-NMR spectra and by comparing with those reported in the literature [12]. The spectra of the pure compound were recorded on the Jeol-Ex at 400 MHz and 100 MHz and on FT NMR spectrometers using methanol as solvent.

### Antioxidant activity

#### DPPH radical scavenging activity

The antioxidant activity of kaempferol-3-O-alpha-L-rhamnoside at various concentrations (3.125 μg/ml to 50 µg/ml) was evaluated via the DPPH radical scavenging system using BHT as standard at the same concentration range of the pure compound (3.125 to 50 µg/ml). Briefly, 3 ml of 0.04% DPPH in methanol was mixed with the compound solution, incubated in dark for 30 min and finally absorbance was measured at 517 nm [13]. The experiment was repeated in triplicate manner and mean absorption was taken to calculate percentage of DPPH radical scavenging activity as follows-

$$\%\;\mathrm o\mathrm f\;\mathrm S\mathrm c\mathrm a\mathrm v\mathrm e\mathrm n\mathrm g\mathrm i\mathrm n\mathrm g=\frac{(\mathrm A0-\mathrm A1)}{(\mathrm A0)}\times100$$

where, A_0_-absorbance of blank.

A_1_- absorbance of pure compound.

IC_50_ (conc. in μg/ml) value required for 50% scavenging of DPPH was calculated from the graph plotted for the % of scavenging against concentration of the sample.

### Antioxidant assay in human erythrocytes

#### Inhibition assay of AAPH-induced hemolysis

Protective effect of the compound against oxidative damage of erythrocytes induced by AAPH was determined by the method described by Ribeiro et al. [14]. Here, hemolysis was performed with AAPH as free radical initiator. An aliquot of 200 μl of 5% (v/v) erythrocytes suspension in PBS was mixed with 50 μl of compound with different concentrations (10, 25 and 50 μg/ml in PBS pH 7.4). Standard ascorbic acid was used at concentration of 50 μg/ml. To this, 0.5 ml of 50 mM AAPH (dissolved in PBS) was added. The reaction mixture was incubated at 37^0^C for 6 h in time dependent hemolysis. Each 1 h interval sample was withdrawn, diluted with 2 ml PBS and centrifuged the mixture at 4000 rpm for 10 min.The supernatant portion was collected and absorbance was taken at 540 nm. Reference values were determined using the same volume of erythrocytes in a hypotonic buffer (100% hemolysis) and kept under same condition as sample. The hemolysis percentage was calculated using the formula-

$$\%\;\mathrm o\mathrm f\;\mathrm H\mathrm e\mathrm m\mathrm o\mathrm l\mathrm y\mathrm s\mathrm i\mathrm s=\frac{(\mathrm{Absorbance}\;\mathrm{of}\;\mathrm{sample}\;\mathrm{supernatant})}{(\mathrm{Reference}\;\mathrm{value})}\times100$$

#### Measurement of lipid peroxidation of erythrocytes

The effect of the compound kaempferol-3-O-alpha-L-rhamnoside on the inhibition of the production of malondialdehyde (MDA), a by-product of lipid peroxidation, was evaluated by incubation of a human erythrocyte suspension (5%) in 50 mM of the oxidizing agent AAPH [15]. Briefly a solution of three different concentration of the compound (10, 25 and 50 μg/ml) and standard ascorbic acid (50 μg/ml) was mixed separately with 0.5 ml of 50 mM AAPH (dissolved in PBS). The reaction mixture was kept at 37 °C for 6 h for time dependent hemolysis. Each 2 h interval one set of sample was withdrawn and 300 μl H_3_PO_4_ (0.44 M), 750 μl thiobarbituric acid (0.67%) were added to 1 ml reaction mixture and incubated at 95 °C for 1 h. This was then cooled in an ice bath for 10 min and 450 μl trichloroacetic acid (20%) was added. The mixtures were centrifuged at 4000 rpm for 10 min.The supernatant portion was collected and absorbance was taken at 532 nm. A control solution of buffer with same volume of erythrocytes was kept under same condition as sample. MDA level was determined using molar extinction coefficient is 156 mM^−1^ cm^−1^ at Beer Lambert Law and the value was expressed as pmol/g Hb.

MDA level = (Absorbance of sample)/(Extinction co-efficient) X Dilution factor.

### Antioxidant assay on plasmid DNA

#### Protective effect on oxidative DNA damage

Protective effect of the pure compound on DNA (pUC19) damage induced by AAPH was performed according to the Zhou et al. [16]. The compound at the concentration (5, 10, 20, 40 µg/ml) from a stock solution of 2 mg/ml and 1 µg DNA (2 µl) were taken in eppendorf tubes after that 10 µl AAPH was added to each mixture. Gallic acid at concentration of 40 µg/ml was used as standard. The reaction mixture was adjusted to the total volume of 40 µl by PBS and allowed to incubate for 30 min at 37^0^C. After 30 min incubation, 6X loading dye (1:1) were added. The reaction mixtures (10 µl) were electrophoresed on 1% agarose in 1X TAE buffer at 110 V for 40 min. The gel was stained with EtBr solution for 30-45 min, viewed under trans-illuminating UV light and photographed using gel documentation system.

### Anti-tumor activity

#### Animals

Swiss albino mice of either sex, 3–4 weeks of age, weighting between 20 and 25 g were collected from the Jahangir Nagar University, Dhaka, Bangladesh. They were maintained under standard laboratory conditions (temperature 22-28^0^C, humidity 55–3%) with 12 h day-night cycles and also provided with standard dry pellet diet and water.

#### Experimental tumor model

Transplantable tumor (Ehrlich’s ascites carcinoma) cells were obtained from Department of Biochemistry, University of Rajshahi and were maintained in our laboratory in *Swiss albino* mice by intra-peritoneal transplantation.

#### Determination of median lethal dose (LD_50_)

The median lethal dose (LD_50_) value was determined following ‘Up and Down’ method described in OECD guideline 425 [17] by injecting the solution of the compound intraperitoneally in mice at various doses (5, 10, 25, 50, 100, 200 mg/kg) and mortality at the end of the 24 h experiment was recorded. Doses were selected for this anti-tumor study by fixed dose methods and were 25 and 50 mg/kg.

### Studies on in vivo EAC cell growth

The pure compound kaempferol-3-O-alpha-L-rhamnoside at doses of 25 and 50 mg/kg per day were given to every mouse of group-II and III, respectively. Group –IV was treated with anticancer drug vincristine. Treatment was continued for 6 days and on seventh day after EAC cell inoculation, animals were sacrificed. EAC cells were collected by repeated washing with 0.9% saline and viable EAC cells per mouse of the treated groups were compared with untreated control [18]. Here, vincristine was used as standard at a dose of 6.25 mg/kg/day. Vincristine is a chemotherapeutic drug that is poisonous to other cells. When it is introduced into cells it binds to the cancer cell DNA which stops the cell division.

### Morphological appearance of EAC cell

Morphological changes of EAC cells were examined by DAPI (4, 6-diamidino-2-phenylindole) staining after collecting the cells from non-treated EAC-bearing mice and mice treated with kaempferol-3-O-alpha-L-rhamnoside (25 and 50 mg/kg/day) for 7 days. Then visual images were taken using fluorescent microscope. Both fluorescent and optical views were observed.

### Studies on hematological parameters

To assess the hematological parameters, Swiss Albino mice were divided into four groups (*n* = 5). All the animals were injected with EAC cells (0.1 ml of 1.6 × 10^6^ cells/mouse) intraperitoneally except the normal group at the day zero. Group 1 served as the normal control, group II served as the untreated EAC control and group III served as the standard vincristine. Group IV and V were treated with compound kaempferol-3-O-alpha-L-rhamnoside at 25 and 50 mg/kg doses, respectively. On the 12th day, after tumor EAC cell inoculation, hematological parameters (Hemoglobin, RBC and WBC) were measured from freely flowing tail vein blood of each mice of each group [19, 20].

### Statistical analysis

The data were analyzed by one-way ANOVA (analysis of variance) followed by multiple comparisons using Dunnett’s post hoc and LSD test using SPSS software of 20version. All results were represented as mean ± standard deviation (SD). Differences at *p* < 0.05 level were considered to be statistically significant.

## Results

### Spectral characteristic

The ^1^H NMR spectrum (MeOD, 400 MHz) of the compound revealed two signals at δ 6.19 (1H, d, J = 1.6 Hz) and δ 6.36 (1H, d, J = 1.6 Hz). Those are well known H-6, H-8 characteristic signals of a flavonoid glycoside. Another two signals at δ 7.76 (2H, d, J = 8.4 Hz) 6.93 (2H, d, J = 8.4 Hz) are characteristic signals of H-3’& 5’, H-2’ & 6’ in kaempferol-3-O-alpha-L-rhamnoside. The signals at δ 5.36 (d, Overlapped), δ 0.91 (overlapped), 4.21, 3.69 are characteristic signals of 3-*O*-rhamnose. Presence of rhamnose was further confirmed by the anomeric carbon at δ 105.9. From the above mentioned data and by comparison with the literature, the compound (Sz-02) was identified as kaempferol-3-O-alpha-L-rhamnoside.

### Antioxidant activity

#### DPPH radical scavenging activity

The antioxidant activity of the compound evaluated by DPPH radical scavenging is shown Additional file Fig. S1. In this study the IC_50_ value for this compound was found to be 14.6 μg/ml while that of BHT was found to be 5.45 μg/ml.

### Antioxidant assay on human erythrocytes

#### Inhibition assay of AAPH-induced hemolysis

**Fig. 2:**
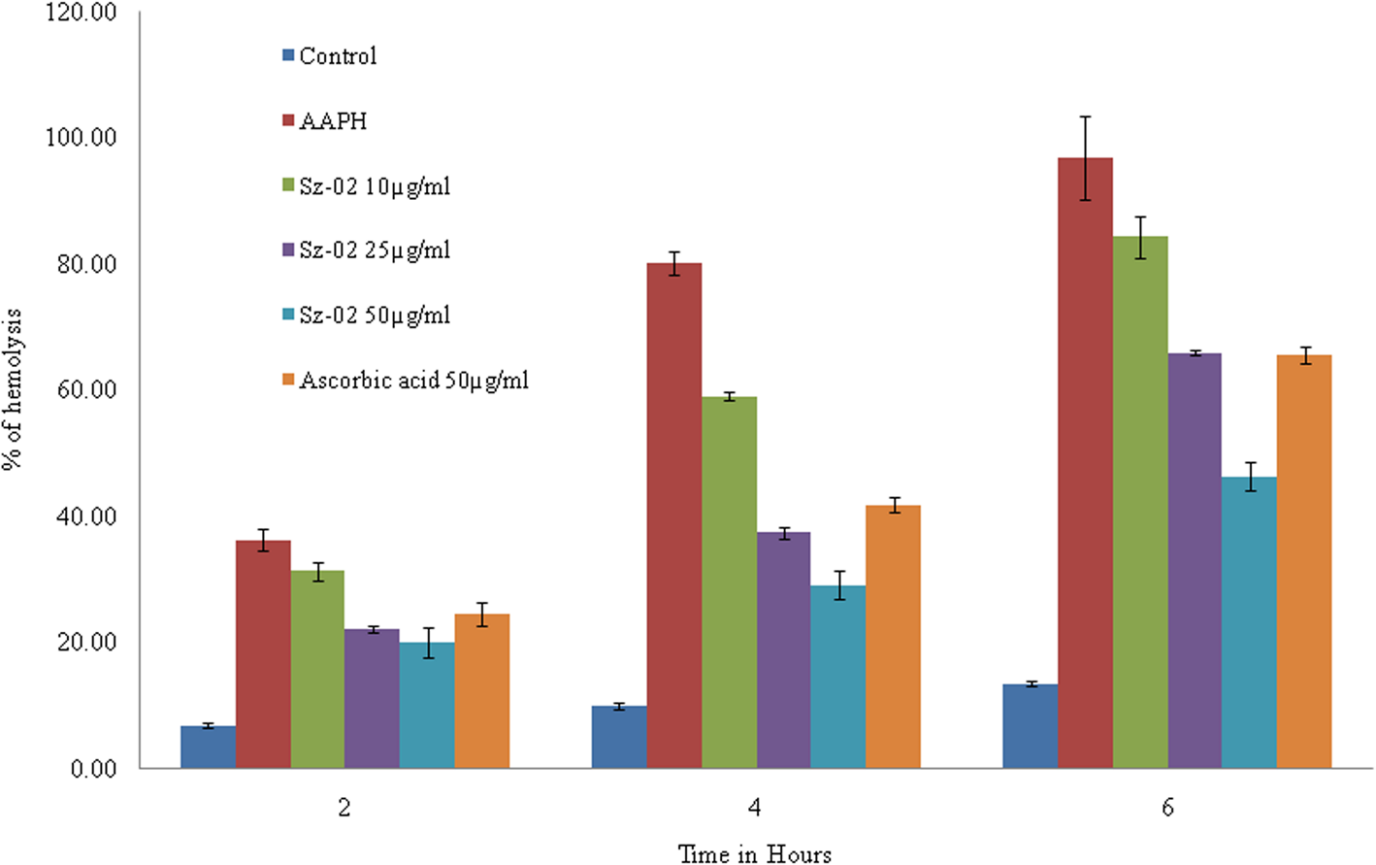
Time course effects of kaempferol-3-O-alpha-L-rhamnoside (Sz-02) and ascorbic acid on AAPH-induced hemolysis on erythrocytes. Erythrocyte suspension at 5% hematocrit was incubated with 50 mmol/l AAPH at 37^0^C in the absence or presence of compound or ascorbic acid at the indicated concentrations. Values are expressed as mean ± standard deviation

Oxidation of erythrocyte membrane by peroxyl radicals generated by AAPH may result in the release of hemoglobin pigment into the medium. The color measurement mediated by hemoglobin at 410 nm serve the level of oxidative damage in cells. Figure 2 showed the effect of kaempferol-3-O-alpha-L-rhamnoside against peroxyl radical-induced hemolysis in erythrocytes. When erythrocytes were incubated with AAPH in air at 37^0^C there was progressive hemolysis after 2 h of incubation and it was 96.73 ± 6.56% after 6 h of incubation. But this hemolytic process was inhibited in the presence of kaempferol-3-O-alpha-L-rhamnoside in a concentration and time dependent manner. After 6 h the percentage of hemolysis was (46.31 ± 2.16%) at maximum tested concentration (50 µg/ml) of the compound whereas 65.44 ± 1.37% haemolysis was observed with ascorbic acid at concentration of 50 µg/ml.

#### Inhibition of malondialdehyde production

**Fig. 3:**
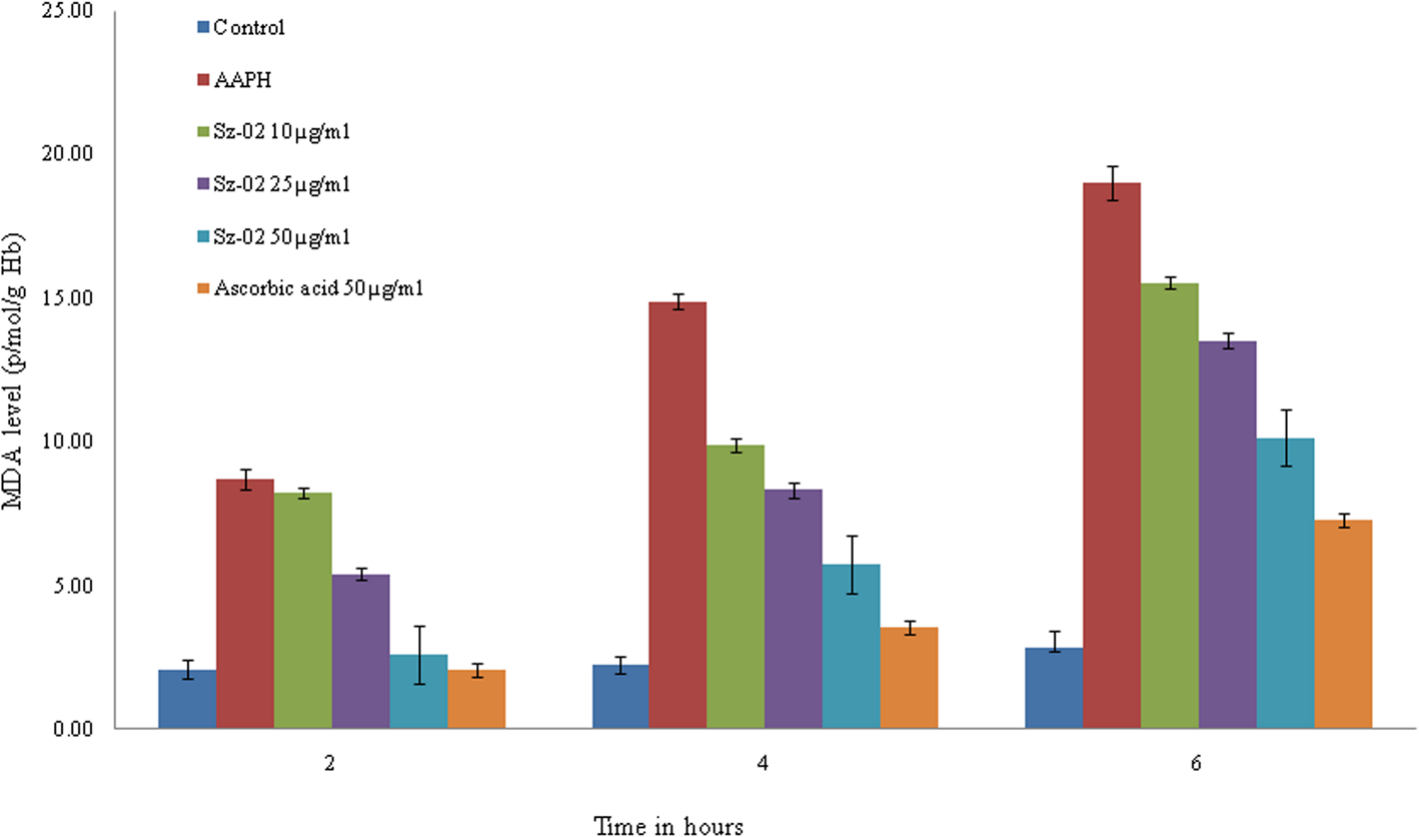
Effect of kaempferol-3-O-alpha-L-rhamnoside (Sz-02) and ascorbic acid on MDA formation in erythrocytes. Erythrocyte suspension at 5% hematocrit was incubated with 50 mmol/l AAPH at 37^0^C in the absence or presence of compound or ascorbic acid at the indicated concentrations. Values are expressed as mean ± standard deviation

AAPH can also cause an increase in lipid peroxidation of erythrocytes. This was evaluated by malondialdehyde (MDA) formation and the results shown in Fig. 3 indicated that AAPH could cause a significant increase in MDA from a range of 8.71 ± 0.34 to 19.90 ± 0.57 nmol/g Hb. But preincubation of erythrocytes with the compound at different concentrations had no significant effect on MDA production. In this case the amount of MDA formation was measured as incubation for 2 to 6 h, ranging from 2.62 ± 0.18 to 10.15 ± 0.22 nml/gHb at maximum concentration of the compound. In case of standard ascorbic acid MDA formation was from 2.08 ± 0.22 to 7.29 ± 0.26 nml/gHb.

#### Antioxidant assay on plasmid DNA

**Fig. 4:**
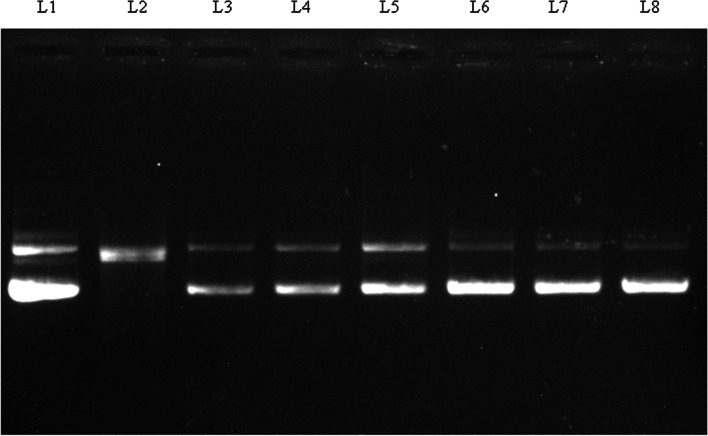
Agarose electrophoretic pattern of pUC19 plasmid DNA with and without the treatment of pure compound and standard gallic acid. Lane 1: untreated DNA; Lane 2: DNA treated with AAPH; Lane 3: DNA + AAPH + 5 µg compound; Lane 4: DNA + AAPH + 10 µg compound; Lane 5: DNA + AAPH + 20 µg compound; Lane 6: DNA + AAPH + 40 µg compound; Lane 7: DNA + AAPH + 50 µg compound; Lane 8: DNA + AAPH + 50 µg gallic acid

The ability of kaempferol-3-O-alpha-L-rhamnoside to protect oxidative damage of DNA was evaluated by analyzing the band pattern of pUC19 DNA on agarose gel as shown in Fig. 4. Lane-1 shows DNA in the native supercoil form, whereas lane-2 treated with AAPH, the super coil form has been converted in open circular DNA. Addition of the compound in lane 3, 4, 5, 6 and 7 at concentration of 5, 10, 20, 40 and 50 µg/ml, respectively prevent the formation of circular form of plasmid DNA in a dose dependent manner. The compound showed the same effect at 40 µg / ml as the standard gallic acid at 50 µg / ml.

### Anti-tumor activity

#### Studies on EAC cell growth inhibition

*In-vivo* anti-tumor activity of kaempferol-3-O-alpha-L-rhamnoside against EAC cell bearing mice was assessed by the parameters such as viable EAC cell (% inhibition in cell growth). The average number of viable tumor cells per mouse of untreated EAC control group was found (7.51 ± 0.83) x 10^7^cells/ml. Effects of the compound on EAC cells growth after tumor inoculation are shown in Table 1. Treatment with kaempferol-3-O-alpha-L-rhamnoside resulted in significant reduction of cell growth *in-vivo*. The percentage of cell growth inhibition of this compound was 37.17 ± 7.94% and 70.89 ± 6.62% at doses of 25 and 50 mg/kg, respectively, whereas standard drug vincristine showed 77.84 ± 6.69%.

**Table 1:** Effect of kaempferol-3-O-alpha-L-rhamnoside on viable Ehrlich ascites carcinoma (EAC) cell growth

Group	Treatment	Viable EAC cells on day 7 after inoculation (× 10^7^ cells/ml)	Percentage (%) cell growth inhibition
1	EAC Cell	7.51 ± 0.83	
2	EAC + Sz-02 (50 mg/kg)	2.16 ± 0.38*	70.89 ± 6.62*
3	EAC + Sz-02 (25 mg/kg)	4.674 ± 0.28*	37.17 ± 7.94*
4	Vincristine (6.25 mg/kg)	1.64 ± 0.51*	77.84 ± 6.89*

Data are expressed as mean ± SD (*n* = 5); Analysis of variance followed by LSD and Dunnett’t post hoc test (IBM-SPSS/20)

^*^P < 0.05: Significance difference with respect to EAC control

#### Morphological changes of EAC cell

**Fig. 5:**
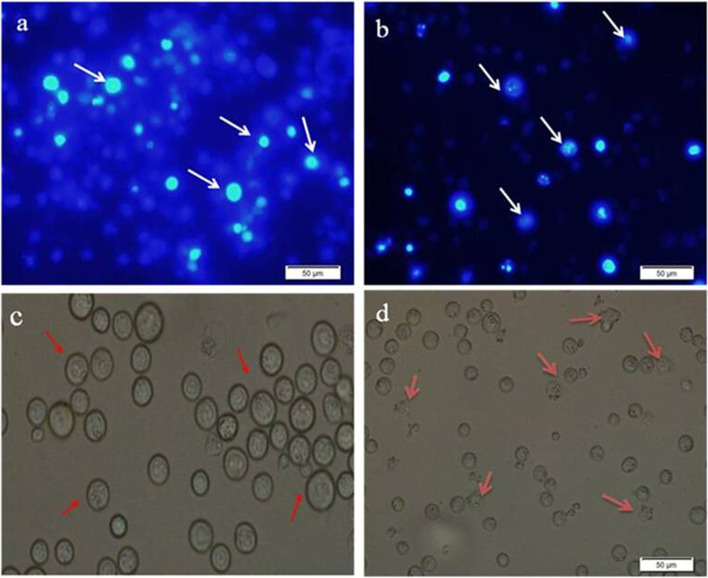
(**a**-**b**) Fluorescence and (**c**-**d**) optical microscopic observation of EAC cells for control mice and treated mice. (**a**) and (**b**) represent as fluorescence microscopic view of control and pure compound treated mice cell where (**c**) and (**d**) express the optical microscopic view of control and pure compound treated cells, respectively. Normal cell with round shape nucleuses appeared in control group indicated by white and red arrow in (**a**) and (**c**) whereas the mice treated with pure compound, condensed nucleus and fragmentation of cells (apoptotic characteristics) were found (**b**) and (**d**) indicated by white and red arrow, respectively

Morphological changes of EAC cells were examined by DAPI staining after collecting the cells from non-treated EAC-bearing mice and mice treated with kaempferol-3-O-alpha-L-rhamnoside (25 and 50 mg/kg/day) after 7 days. EAC cells nuclei were round, regular, and homogeneously stained with DAPI in control group (solvent treated) as shown in Fig. 5. Apoptotic morphologic alterations such as membrane and nuclear condensation were noted in Kaempferol-3-O-alpha-L-rhamnoside treated EAC cells. These results indicated treatment with this compound could induce apoptosis in EAC cells.

#### Studies on hematological parameters

Hematological parameters of untreated EAC cell bearing mice on the day 12 were showed significant (*P* < 0.05) changes when compared to normal mice (Table 2). The total WBC count was found to increase with a reduction in the hemoglobin content and total RBC count. At the same time interval, treatment of compound (25 and 50 mg/kg) could brought back these altered parameters to normal values. The overall results of this study clearly demonstrated the anti-tumor activity of the compound against EAC.

**Table 2:** Effect of kaempferol-3-O-alpha-L-rhamnoside on blood parameters of tumor bearing and normal mice

Parameters	Normal	Control	EAC + Kaempferol (50 mg/kg)	EAC + Kaempferol (25 mg/kg)	EAC + Vincristine (6.25 mg/kg)
Hgb (g/dl)	13.89 ± 0.08	9.20 ± 0.26*	12.99 ± 0.33^#^	10.24 ± 0.48^#^	13.52 ± 0.28^#^
RBC (× 10^9^cells/ml)	6.34 ± 0.32	1.85 ± 0.15*	3.35 ± 0.165^#^	3.2 ± 0.118^#^	6.05 ± 0.52#
WBC (× 10^6^cells/ml)	8.23 ± 0.135	121.60 ± 1.81*	28.80 ± 0.72^#^	51.47 ± 0.59^#^	17.87 ± 0.48#

Data are expressed as mean ± SD for five animals in each group. Analysis of variance followed by LSD and Dunnett’t post hoc test (IBM-SPSS/20)

^*^P < 0.05: against normal group and

^#^*P* < 0.05: against EAC control group

## Discussion

*Pithecellobium dulce* is used as astringent in dysentery, abortificient, antidiabetic, anticonvulsant, antiulcer, larvicide dermatitis, eye inflammation, indigestion, intestinal disorder, ear ache, leprosy and tooth ache. Phytochemical investigation of bark and leaves had revealed the presence of *β*-sitosterol, saponin glycosides, oleanolic and echinocystic acids as sapogenins, echinocystic acid, bisdesmoside, dulcin, triterpenoids, acylated triterpenoid saponin, flavanoids, saccharrides, long chain aliphatic hydrocarbons, and tannins [21, 22]. In our study, kaempferol-3-O-alpha-L-rhamnoside; a flavonoid, commonly known as afzelin was isolated from leaves extract of *P. dulce*. The spectral characteristics of this isolated compound showed similarities with previously reported a flavonoid like glycoside [23]. Before final extraction the leaves were preliminarily extracted with n-hexane to remove chlorophyll present in leaves that could interfere with the separation of other constituents and this extract was discarded. In order to extract different phenolic/flavonoid compounds from plants with a high degree of accuracy, various solvents of differing polarities must be used. Moreover, highly polar solvents, such as methanol, have a high effectiveness as antioxidants [24]. So we choose methanol for final extraction and then fractionated with different solvents of different polarity.

To know the biological activity of the isolated compound, we determined in vitro free radical scavenging and protection of oxidative damage on DNA and human erythrocytes as well as anti-tumor activity on EAC cells and showed promising results. Antioxidants fight against free radicals and protect us from various ailments [7]. Isolation and identification of many natural antioxidants from different plant materials have been studied. DPPH radical is one of the most common and stable chromogens used to estimate antioxidant activity of biological materials in a relatively short time [25]. In our previous study we found that among the different fractions of crude methanolic extract the ethylacetate fraction showed strong DPPH radical scavenging activity with IC_50_ of 9.44 µg/ml. Here, the DPPH radical scavenging power (IC_50_ = 14.6 μg/ml) of the isolated compound from this fraction suggested that the isolated compound may be a major component of the EAF responsible for this radical scavenging activity.

For a better understanding of the antioxidant activity of the compound, we used a human erythrocyte to measure hemoglobin oxidation and lipid peroxidation inhibition. Free radicals attacking of erythrocyte membrane components (proteins and lipids) cause the alteration of membrane structure and function leading to hemolysis. The peroxy radicals generated from AAPH are capable of inducing lipid peroxidation and protein damage [26]. In the present study, the incubation of erythrocyte together with AAPH led to remarkable hemolysis that was consistent with previous findings [27]. The present results clearly show that the pure compound has high capacity to prevent the oxidative damage induced in the erythrocyte membrane and to minimize the lipid peroxidation. Lipid peroxidation from AAPH induced radical results in the production of MDA which is responsible for cellular damage and associated with many of pathological events [28]. Since the compound showed ability to protect oxidation of erythrocyte membrane so the inhibition of lipid peroxidation was also expected and we found that the compound could inhibit the malondialdehyde formation in a dose dependent manner. Therefore, the protective effects of erythrocyte membrane and inhibition of MDA production of this pure compound may result from diminishing peroxyl radicals generated from AAPH during the incubation period as it was also effective in scavenging of DPPH free radicals.

Cellular DNA damage may cause alteration of replication and transcription causing cell death or mutations. Such modification of DNA is responsible for aging and various diseases, including Alzheimer, Parkinson and cancer etc. So, study on DNA damage protection is important. Oxidative DNA protective activities of flavonol type glycoside have been previously reported by other researches [29]. Here we reported the effectiveness of the isolated pure compound from leaves extract to prevent AAPH induced oxidative damage of DNA. AAPH converted the super coiled DNA strand into open circular [30]. The compound could significantly inhibit the formation of the open circular form in a dose dependent manner as compared to standard gallic acid (Fig. 4). This result demonstrated the DNA damage inhibition potential of this compound, therefore, can be used in cancer prevention.

So, next we investigated the role of this compound in cell growth inhibition on EAC bearing Swiss albino mice. The prolongation of the life span & reduction of tumor weight of cancer bearing mice is a very important and reliable criterion for judging the potency of any drug as anticancer agent [31]. The effectiveness of the compound against EAC cell bearing mice has further been verified by monitoring the change in hematological and biological parameters. This study indicates that the number of cell growth decreased and number of apoptotic cells increased significantly at different doses. Previously reported the effect of kaempferol-3-O-alpha-L-rhamnoside on the viability of MCF-7 and HC-04 cells was evaluated. The treatment of cancer (MCF-7) and non-cancer (HC-04) cell lines with kaempferol-3-O-alpha-L-rhamnoside resulted in a dose-dependent inhibition of cell growth, [32] which also support our findings. Anemia may occur during cancer chemotherapy. In our study, reduction in RBC or % in hemoglobin in tumor bearing mice may occur which is mainly due to iron deficiency or hemolytic or myelopathic conditions. Kaempferol-3-O-alpha-L-rhamnoside could significantly recover the hemoglobin content; RBC and WBC cell count that indicates the protective action of this compound (Afzelin) on the haemopoietic system. All these are measured are very important aspects in justifying the effectiveness of a compound in cancer chemotherapy.

## Conclusion

This study is the first to evaluate the antioxidant activity of kaempferol-3-O-alpha-L-rhamnoside present in the leaves of *P. dulce* in a comprehensive manner employing a range of assays. The results of the present study show that the pure compound possesses antioxidant activity evidenced by various methods as well as cell growth inhibition on EAC cells. The study showed the DNA damage inhibition potential of the compound, which could be used in cancer prevention. Further work should be carried out to establish the mechanism of the above activity and to determine its safety.

## Supplementary Information

**Additional file 1**: **Figure S1**: DPPH radical scavenging activity of the pure compound and standard BHT.

## Data Availability

All data generated or analyzed during this study are included in this published article (and its supplementary information files).
