# Supplementary material for: Anti-tumor and antioxidant activity of kaempferol-3-O-alpha-L-rhamnoside (Afzelin) isolated from Pithecellobium dulce leaves
Source: BMC Complement Med Ther. 2022 Jun 22;22:169. doi: 10.1186/s12906-022-03633-x (PMC9219166; doi:10.1186/s12906-022-03633-x)
Supplement: Supplementary file 1 — Additional file 1: Figure S1: DPPH radical scavenging activity of the pure compound and standard BHT. [file 12906_2022_3633_MOESM1_ESM.docx]

Additional file: Figure S1: DPPH radical scavenging activity of the pure compound and standard BHT
